# The kinetics and mechanism of the uranium hydride - water vapour system under ambient conditions

**DOI:** 10.1038/s41598-020-66462-3

**Published:** 2020-06-11

**Authors:** A. Banos, T. B. Scott

**Affiliations:** University of Bristol, Interface Analysis Centre, School of Physics, HH Wills Physics Laboratory, Tyndall Avenue, Bristol, BS8 1TL United Kingdom

**Keywords:** Surface chemistry, Materials for energy and catalysis, Nuclear fuel, Nuclear waste

## Abstract

This work investigated the reaction of uranium hydride powder with saturated water vapour at 25 °C. Two corrosion experiments were conducted one with deionised water (H_2_O) and one with deuterated water (D_2_O). The kinetics of the reaction were measured through gas generation method while concurrent residual gas analysis (RGA) allowed better understanding of the oxidation mechanism governing the system. From the analysis, it was found that the kinetics of the reaction are robust initially, followed by quasi-linear decelerating regime indicative of a ‘shrinking core’ type oxidation behaviour. The extent of the reaction (conversion to UO_2_) was lower in comparison to other works. The reaction remained incomplete bolstering the case of UH_3_ persistence in legacy wastes. Through interpretation of the gas analysis data, a mechanism for the uranium hydride water reaction was suggested.

## Introduction

The Sellafield legacy ponds and silos consist of four plants which were historically used for the interim storage of unconditioned waste awaiting to be reprocessed or prepared for long-term storage and disposal^1,2^. Intermediate level waste (ILW), mainly comprised of uranium-contaminated materials like Magnox cladding, etc. and radioactive sludge have been accumulated in these plants for over six decades to keep them safely isolated from the environment^1,3^. Under a water environment uranium oxidises to produce uranium dioxide (UO_2_), and H_2_ gas (Eq. 1). In an enclosed environment, H_2_ can be trapped in the vicinity of U and in high concentrations, may react with it to produce uranium hydride (UH_3_) (Eq. 2)^4,5^. Such reaction may be regarded as unwanted since uranium hydride behaves pyrophorically under sudden exposure to air and under certain conditions (large quantity and high surface area)^6^.

1 $${\boldsymbol{U}}+2{{\boldsymbol{H}}}_{2}{\boldsymbol{O}}\to {\boldsymbol{U}}{{\boldsymbol{O}}}_{2}+2{{\boldsymbol{H}}}_{2}$$

2 $$2{\boldsymbol{U}}+3{{\boldsymbol{H}}}_{2}\to 2{\boldsymbol{U}}{{\boldsymbol{H}}}_{3}$$

There has been an ongoing controversy between different research groups on whether the solid corrosion products arising will contain UH_3_^4,5,7–19^ or not^20–23^, in addition to UO_2_, and if so, how much is likely to be present and in what distribution.

Recently, the kinetics of the uranium-water corrosion reaction was examined under immersed and contained conditions for prolonged periods (up to ~60 days)^4,5^. Analysis of the long-duration experiments showed that under certain conditions bulk-UH_3_ formation can and will occur on a uranium sample. The parameters affecting the quantity of UH_3_ forming in such a system were also verified^4,5^. It was found that the ratio of UH_3_ to the oxide corrosion products decreased as the reaction period and temperature of reaction increased. The parameters affecting the location of the hydrides and hydriding behaviour of the metal were also examined^24–27^. The abundance of water in the uranium-water-hydrogen system has led us to assume that an additional corrosion process is occurring among others in this complex ternary system, the oxidation of UH_3_ with water^4,5^. This reaction is a highly favourable process as it turns pyrophoric UH_3_ to UO_2_, which, depending on surface area may be considered less reactive.

There is only a limited amount of literature available on the oxidation of UH_3_ with water^11,22,28–35^. According to the literature, the oxidation proceeds through the following exothermic reaction:

3 $$2{\boldsymbol{U}}{{\boldsymbol{H}}}_{3}+4{{\boldsymbol{H}}}_{2}{\boldsymbol{O}}\to 2{\boldsymbol{U}}{{\boldsymbol{O}}}_{2}+7{{\boldsymbol{H}}}_{2}$$

The reaction kinetics of UH_3_ with water vapour and liquid water are regarded to be the same^33,34^. Through the reaction of 0.2–1 g of UH_3_ with liquid water, Beethan *et al*.^30^ observed bubbles, ascribed to H_2_, being released to the water. The reaction was very rapid initially and slowed after 2–5 h. Spedding *et al*.^31^ found that the sample mass to water ratio played an important role in the reaction, with small amounts of water only reacting with UH_3_ regionally and not self-sustaining the reaction. Baker *et al*.^11^ reacted UH_3_ with water vapour and observed the kinetics to decrease after an early accelerating stage, consuming 15–20% of the hydride after two weeks of reaction at environmental conditions. The reaction only reached 83% of completion at 100 °C, under saturated conditions, suggesting that the reaction kinetics slow to an almost negligible rate. Baker’s findings were verified in a recent work by Goddard *et al*.^33,34^, who observed this same termination stage after 80–90% of the sample was consumed.

There are a number of parameters affecting the reaction kinetics and also the extent of UH_3_ oxidation. Baker *et al*.^11^ found that the extent of UH_3_ transformation to UO_2_ was significantly increased with increasing temperature. Beetham suggested that water vapour pressure has a P^0.2-0.6^ dependence with the rate, while Goddard *et al*.^33,34^, after extrapolation of the rate to condensation conditions, suggested a square root dependence. However, the fraction of UH_3_ reacted with water to form oxide was found to decrease with increasing water vapour pressure^11^. The temperature of UH_3_ formation greatly affected the kinetics of UH_3_ oxidation^33,34^, with low temperatures of hydride formation resulting in subsequently enhanced oxidation kinetics^33,34^, meaning the hydride material was more reactive. Finally, oxygen additions in water vapour were observed to accelerate the reaction^32–34^, which is in direct contrast to what is well observed for the uranium-water reaction^19,21,23,36–43^.

In this work, the reaction of UH_3_ powder with water vapour under saturated environmental conditions will be examined. These conditions are most relevant to storage conditions at Sellafield. Two types of water vapour will be used for the reactions, normal de-ionised/purified water and ‘heavy’ water (D_2_O). Analysis of the evolved gases will be performed using residual gas analysis to better understand the oxidation mechanism operating in the system.

## Results

### *In-situ* UH_3_ formation and analysis by hot-stage XRD

The X-ray diffractometer, with the built-in gas rig (Fig. 2), was employed to hydride the surface of the sample *in-situ* and determine the chemical phase transformation occurring on the surface of the metal. Identical conditions were used for surface hydriding as for the experiment described in Section 1.2.2. The analysis was conducted to mimic the conditions of the reaction cell, producing newly-formed UH_3_, and then immediately characterising it prior to any measurable oxidation. Preparation elsewhere and then transfer to the XRD would have resulted in a deleterious exposure of the hydride to air; hence this was the only possible experimental approach. No crystallite or particle size measurements were conducted since transformation of uranium was only occurring on the surface and analysis was only performed to confirm that UH_3_ was formed. During hydrogen exposure of the metal sample, the XRD continually probed the sample and after the first UH_3_ intensity peak was observed the reaction was halted and the cell volume evacuated. Subsequent detailed XRD analysis was performed under vacuum using a Cu K_α_ source between 25–50 ° 2θ with 0.05 ° steps and five second dwell time. Figure 3 illustrates the XRD spectra recorded after hydride formation (formed at 500 mbar of H_2_, 240 °C). A thick hydride layer was formed on the surface (linear reaction stage - reaction front formation) since the XRD spectra showed only the five main hydride peaks.

**Figure 1:**
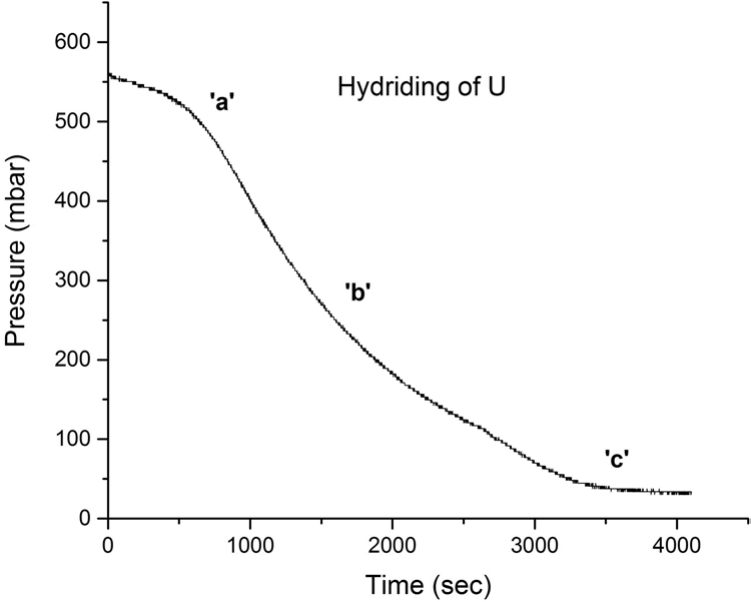
Hydriding of Magnox-U at 240 °C, 550 mbar H_2_.

**Figure 2:**
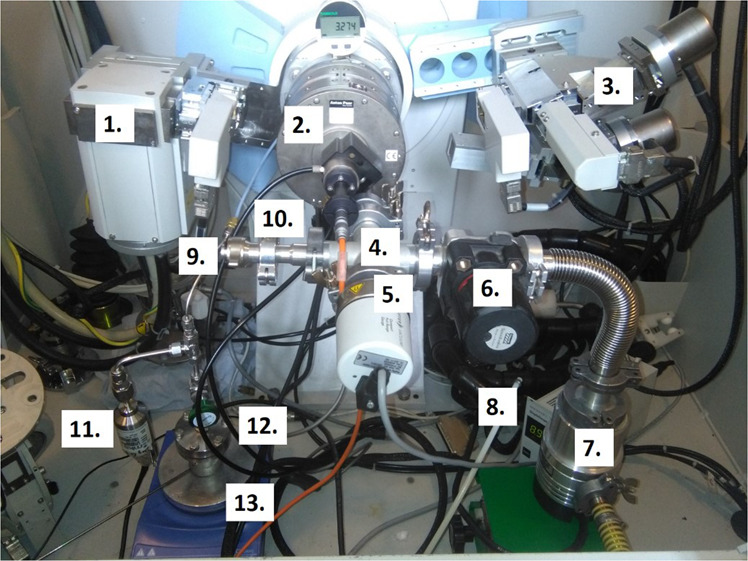
Experimental set-up developed for hot-stage X-ray diffraction (XRD) hydriding analysis. The set-up is composed of: (1) X-ray tube; (2) HTK 1200 high temperature oven chamber; (3) X-ray detector (receiving side); (4) 22 mm pipe cross-piece; (5) Full range pressure gauge; (6) Speed valve; (7) Turbo pump (backed-up by a roughing pump); 8) Full range gauge controller and digital display; (9) Bleed-valve; (10) Fitted high temperature sample stage; 11) 0 - 6895 mbar pressure transducer; (12) Source hydrogen) bed; (13) High temperature hot-plate.

**Figure 3:**
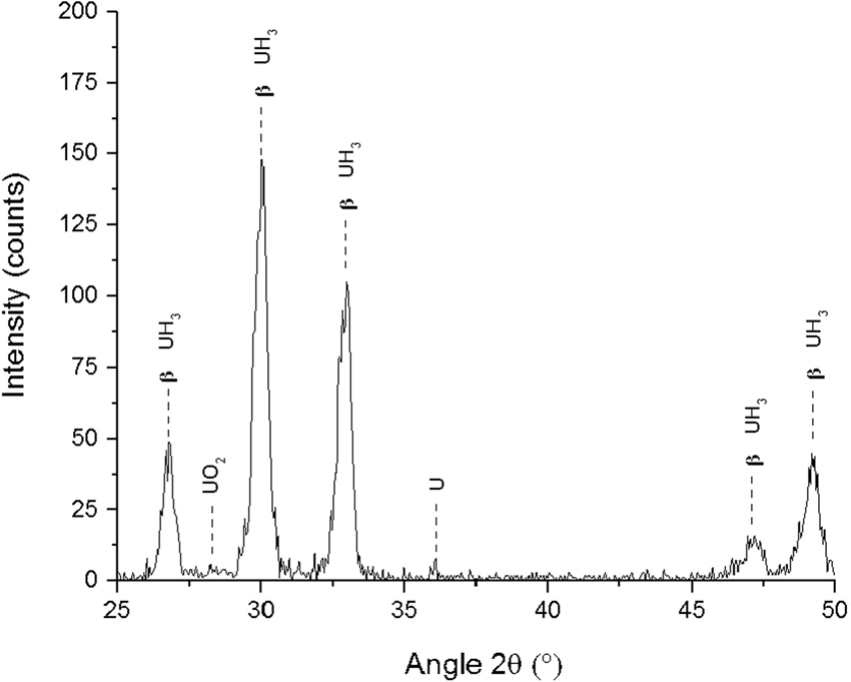
Raw X-ray diffraction (XRD) spectra for a reference sample hydriding at 240 °C, 500 mbar H_2_. The analysis was performed with a Cu K_α_ source at 8 keV, between 25 and 50 ° angle 2θ, 0.05 step and 5 sec dwell time.

### Surface area analysis

The surface area of the sample was found to be ~1 m^2^.g^−1^. In the literature, the reported surface area of UH_3_ powder used in experimental studies has ranged from 0.3–1.7 m^2^.g^−1^ ^11,44–49^. The surface area is considered to be mainly affected by three parameters, the temperature of formation, the mass of the sample and the number of hydriding cycles applied to the material. The surface value of this work was higher than Totemeier *et al*.^46^ who calculated a surface area of 0.5–1 m^2^.g^−1^ and lower than from Goddard *et al*.^33^ who measured a surface are of 1.24 m^2^.g^−1^ (temperature of formation 80 °C).

### Reaction rate determination

As the experimental cells has been thoroughly leak tested prior to the experiment, any observed pressure increase in the cell headspace, over time, was solely ascribed to H_2_ gas generation from the oxidation of UH_3_, according to Eq. (3). The measured pressure was converted to moles of produced H_2_ gas using the ideal gas law through Eq. (4):

4 $${{\boldsymbol{n}}}_{{{\boldsymbol{H}}}_{2}}=({{\boldsymbol{P}}}_{{{\boldsymbol{H}}}_{2}}{\boldsymbol{V}})/({\boldsymbol{R}}\,{\boldsymbol{T}})$$

where P_H2_ (converted in atm) is the measured pressure of H_2_ gas, V (in L) is the volume of the system, R is the ideal gas constant and has the value 0.082057 L. atm. mol^−1^. K^−1^ and T (in K) is the temperature of the cell. Through Eq. (3), the moles of generated H_2_ were then converted to milligrams of reacted UH_3_. If complete conversion of UH_3_ was assumed from Eq. (3), a theoretical expected value of mg UH_3_ could be derived for each experiment. Subsequently the complete temporal data set was processed to calculate and plot the reaction rate as a function of increasing time from the onset of oxidation. The reaction rate was presented as the percentage conversion of UH_3_ to UO_2_ per unit time (mg of _reacted_ UH_3_.mg_theoretical_ UH_3_^−1^.h^−1^).

### UH_3_ + H_2_O experiment

**Figure 4:**
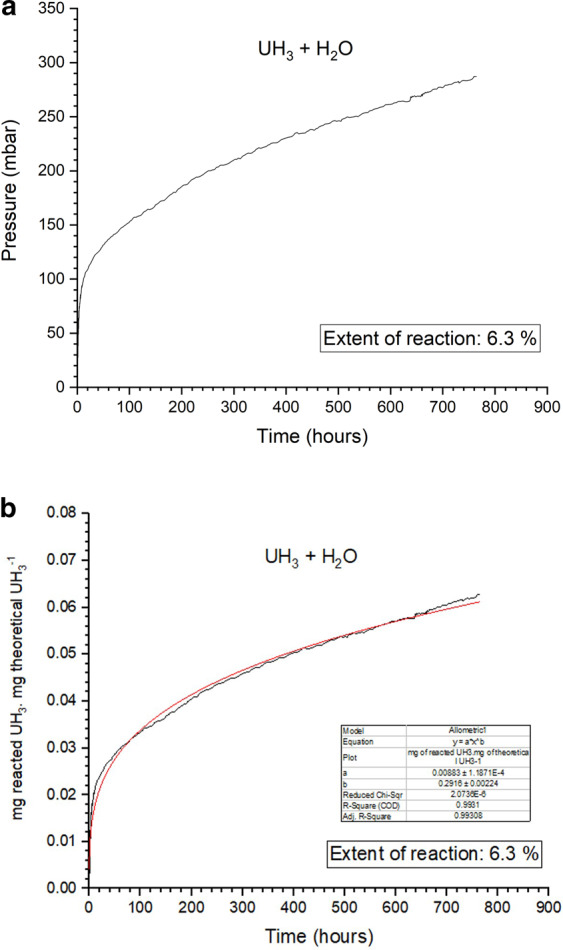
The oxidation of UH_3_ with H_2_O vapour for ambient conditions (25 °C, 31 mbar H_2_O). On (**a**) the raw pressure generation data over time are displayed and on (**b**) the rate is defined as the proportion of the total UH_3_ reacted to the theoretical of 2227.9 mg of UH_3_ over time.

A 2.2 g uranium hydride powder sample was reacted with water vapour under ambient conditions (~25 °C, ~31 mbar H_2_O) in this experiment. Figure 4a,b illustrate the raw pressure generation data over time and the arising reaction plot, respectively. After non-linear fitting it was found that the reaction line profile could be expressed adequately by the classical Freundlich model as in Eq. (5):

5 $${\boldsymbol{y}}={\boldsymbol{a}}{{\boldsymbol{x}}}^{{\boldsymbol{b}}}$$

with a= 0.0088 being a coefficient and b= 0.2916 being the power of x. The Freundlich model generally represents the isothermal variation of adsorption of a quantity of gas adsorbed by unit mass of solid adsorbent with pressure^50^. It can also represent the amount absorbed per unit mass of absorbate. Here, the formula revealed that the rate of conversion of UH_3_ to UO_2_ was initially very robust and was governed by how fast water was adsorbing in the surface to react with ‘fresh and non-oxidised hydride. The conversion rate continues to drop significantly over time with the UH_3_ particles following a ‘shrinking core’ oxidation model where the UH_3_ core is becoming smaller in size due to reaction over time. Uranium oxide acts as a protective layer which inhibits diffusion of the reactive agents to reach the oxide-hydride interface. This latter effect combined to the reduction of the reaction surface area govern the reaction kinetics at this later stage. Over a ~764-hour time period 6.3% of the starting UH_3_ mass had been converted to UO_2_, which marked the maximum extent of the reaction.

### UH_3_ + D_2_O experiment

**Figure 5:**
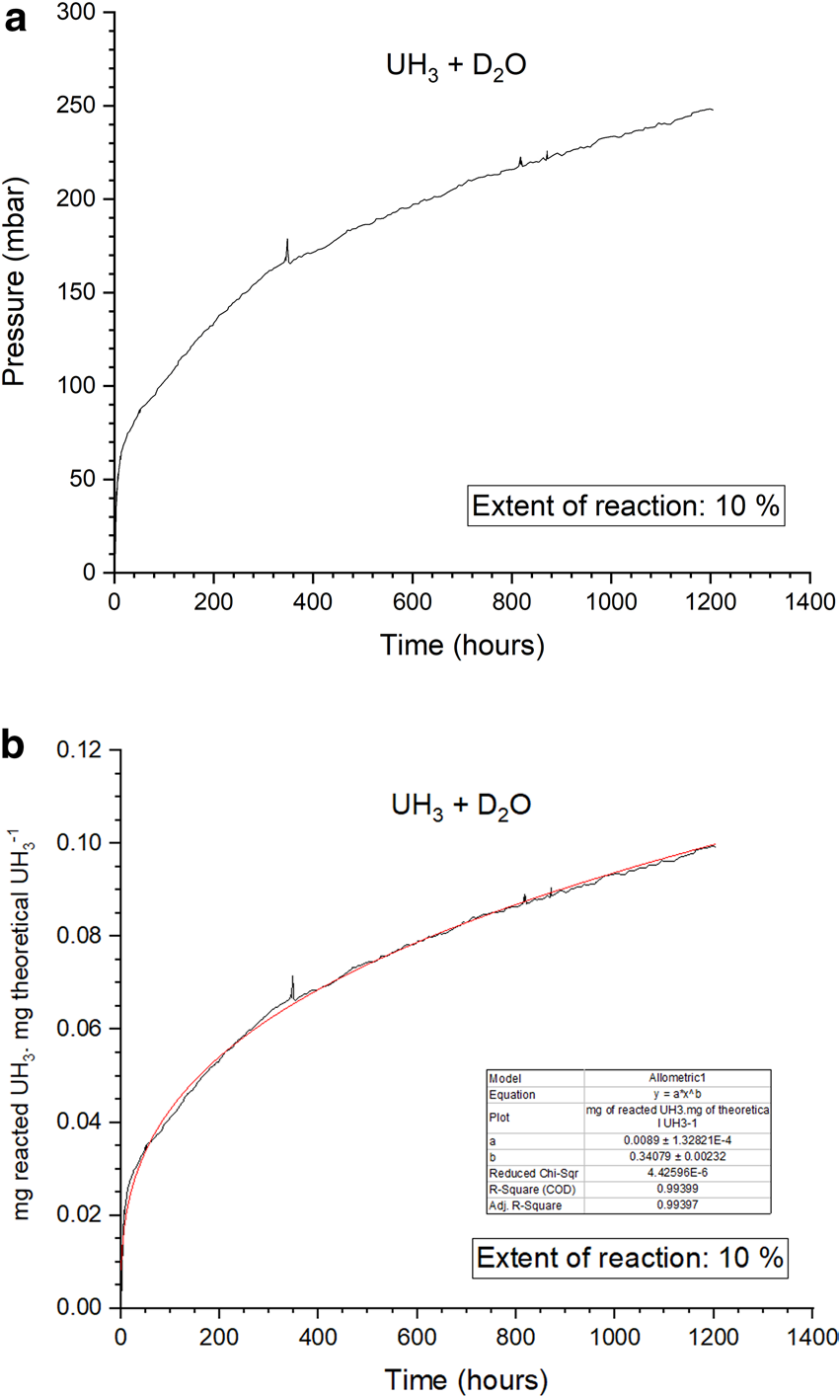
The oxidation of UH_3_ with D_2_O vapour for ambient conditions (25 °C, 31 mbar D_2_O). On (**a**) the raw pressure generation data over time are displayed and on (**b**) the rate is defined as the proportion of the total UH_3_ reacted to the theoretical of 1215.4 mg of UH_3_, over time.

A 1.2 g powder sample was prepared and reacted for this second experiment. Figure 5a,b show the derived pressure data and the corrosion progression over time for UH_3_ oxidizing with saturated D_2_O vapour at ~25 °C. The same behaviour was observed here as for the sample of the UH_3_ + H_2_O experiment. The reaction rate line profile also obeyed to Eq. (5) with the following parameters being derived from non-linear fitting:

a = 0.0089 being a coefficient and b = 0.3408.

The percentage extent of UH_3_ transformation was ~10% for 1204 h of oxidation.

### Reaction rate comparison

The two arising data sets showed very similar behaviours and the derived rates were very closely comparable. Table 1 integrates some of the parameters from both experiments. The rates were found to switch from the early initial robust stage to a gradual decelerating stage at ~41.1 h and ~43.5 h for UH_3_ + H_2_O and UH_3_ + D_2_O, respectively. The rate of the H_2_O experiment at this stage was ~44% slower than the one of the D_2_O experiment. The reaction period between the two experiments was very different since the H_2_O experiment was stopped after 763 h and the D_2_O experiment after 1204 h. This affected the extent of the reaction which also varied between the experiments (H_2_O experiment = 6.3%, D_2_O experiment = 10%). Of course, the extent of reaction could also be affected by the rate of conversion/reaction. To compare the reaction kinetics between the experiments, the conversion rate was calculated at a random point by using Eq. (5). For a selected value of x= time= 600 h, the reaction rate was 0.000095 and 0.000131 mg_of reacted_ UH_3_.mg_theoretical_ UH_3_^−1^.h^−1^ for the UH_3_ + H_2_O and UH_3_ + D_2_O systems, respectively. Thus, the conversion rate was 38.55% faster on the D_2_O experiment when compared to the H_2_O experiment.

**Table 1:** The parameters of the UH_3_ + H_2_O and UH_3_ + D_2_O experiments.

Experiment	Sample mass (g)	Reaction period (hours)	y= ax^b^	Reaction rate k -at 600 h (mg_of reacted_ UH_3_.mg_theoretical_ UH_3_^−1^.h^−1^)	Extent of reaction (%)
UH_3_ + H_2_O	2.2	~764	a=0.0088, b=0.2916	0.000095	~6.3
UH_3_ + D_2_O	1.2	~1204	a= 0.0089, b= 0.3408	0.000131	~10

In the literature, there was only a limited amount of work reporting the kinetics of this reaction, especially in such a low temperature regime. Baker *et al*.^11^ reported 15–20% of UH_3_ conversion to UO_2_ for a 336 h oxidation time period. Based on the reaction extent, the kinetics were considerably faster in comparison to this work, since for the H_2_O and D_2_O experiments only 4.8% and 6.6% of the hydride reacted over the first 336-hour period. Goddard *et al*.^33^ used TGA analysis to examine the oxidation reaction of UH_3_ over a wide range of temperatures with water and water vapour. They observed the reaction of UH_3_ prepared at 80 °C, oxidising at 30 °C, over saturated conditions. For such a hydride formation temperature (80 °C), the production of both α-UH_3_ and β-UH_3_ would be expected^51,52^. Owing to the different expected reactivity between α-UH_3_ and β-UH_3_ with water they yielded two different rates, one for each component. Through comparison of the reported rate from oxidation of the β-UH_3_ component at 30 °C, it was found that the quasi-linear rate of 0.0004 mg_reacted_ UH_3_.mg_theoretical_UH_3_^−1^.h^−1^ which was suggested in that work is lower than those determined in this work.

### Residual gas analysis

**Figure 6:**
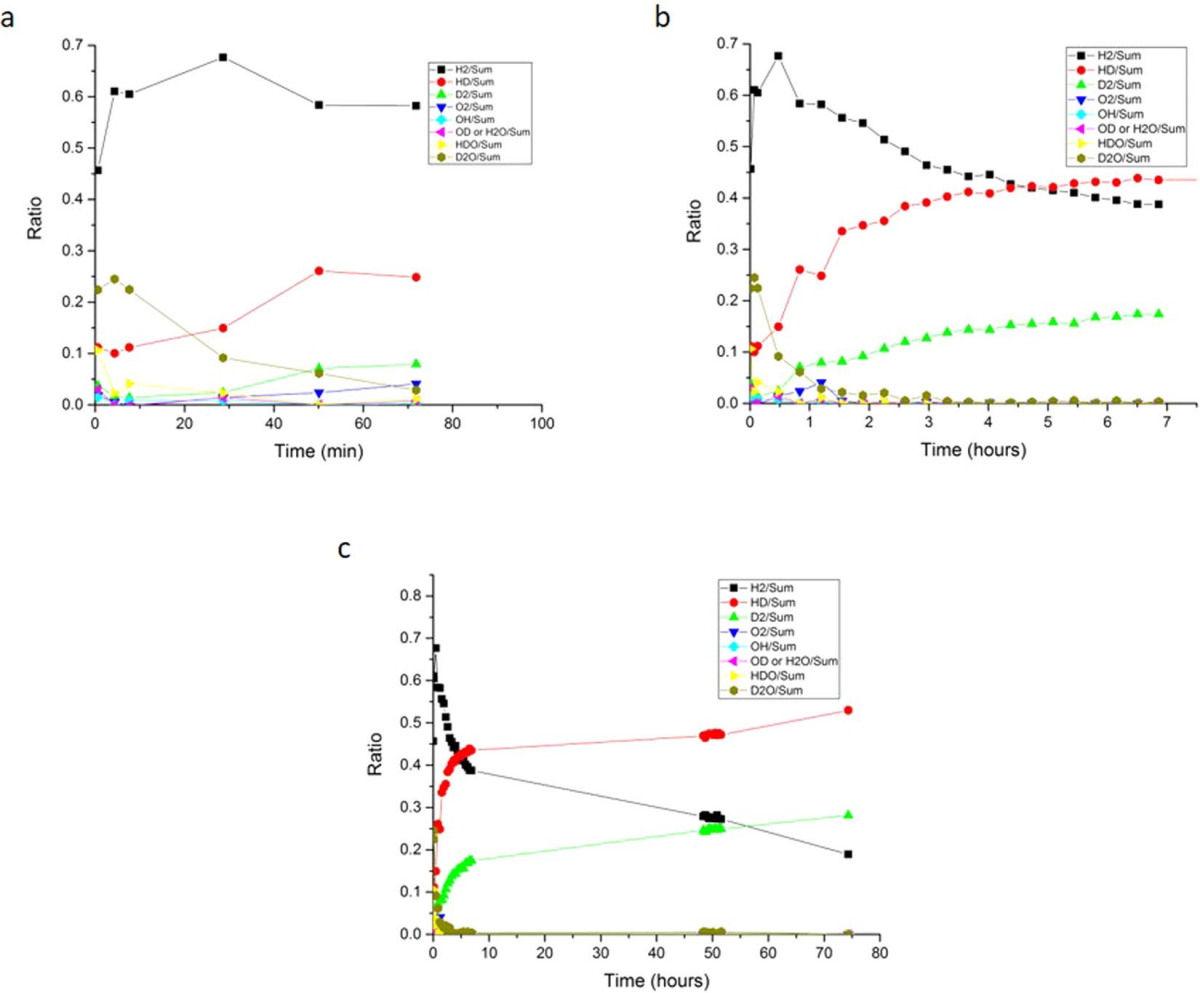
Ratio profile of the relative gas pressure of the evolved gases from oxidation reaction of UH_3_ with D_2_O for (**a**) 1 h (**b**) 7 h of oxidation and (**c**) 75 h of reaction.

RGA analysis of the evolved gases was conducted periodically to gain a closer insight into the corrosion mechanism of UH_3_ oxidation. The method of analysis was described in Section 1.2.5.2. The results from gas analysis on the UH_3_ + H_2_O system showed almost solely H_2_ gas generating in the headspace. Insignificant amounts of H_2_O were also detected. However, not much information about the mechanism of the reaction could be drawn since origination of H^+^ ions could not be resolved, between the UH_3_ and H_2_O. Using isotopic labelling of the water, the path of D^+^ ions derived from water and H^+^ ions from UH_3_ could be traced and the mechanism of oxidation could be verified. For UH_3_ + D_2_O, the headspace gas was analysed with an initial 20-min periodicity for the first 7 h of reaction. Gas sampling and analysis was then performed at ~50 h and ~75 h of reaction. As already explained in Section 1.2.5.2, at least 11 full scans were performed for each time interval where the sampling valve was opened. After background subtraction (blank experiment), the ratio of the relative gas pressure (detected as a specific mass in amu) to the overall gas pressure was derived. Figure 6a–c show the ratios of the detected gases over exposure time. H_2_, HD, D_2_, O_2_ OH, H_2_O or OD, HDO and D_2_O were detected after background subtraction.

Over the first 30 min of oxidation, we observed a sudden increase in the H_2_ gas ratio, concomitant with a decrease in the D_2_O ratio and slight increase in the HD ratio (Fig. 6a). Following the first 30 min, the D_2_O ratio drops to almost zero (background level). Apart from H_2_, HD and D_2_ ratios, all detected gas ratios fell to almost zero/background levels at this stage (Fig. 6b). Interestingly, the H_2_ gas ratio starts to decrease simultaneous to an observed increase of HD and D_2_ ratios (Fig. 6a,b). After this point, H_2_ gas ratio continued to decrease and HD, D_2_ ratios continued to increase parabolically until analysis was ceased (Fig. 6c).

## Discussion

From both corrosion experiments, the measured rates from the quasi-linear decelerating regimes were consistently lower in comparison to the limited amount of data reported in the literature. As already discussed in the introduction the rate of the reaction, after the early initial stages of oxidation, is mainly affected by four different parameters, the temperature of reaction, the water vapour pressure, the temperature of UH_3_ formation and oxygen additions. Higher temperature, higher water vapour pressure and higher oxygen additions would result in enhanced rate kinetics, contrary to the temperature of UH_3_ formation where the effect is the inverse (higher formation temperature leads to decreased rate). In Baker’s work^11^, where the rate of reaction was significantly higher, the conditions of UH_3_ formation were not stated in the paper. It is believed that the temperature of formation was lower than the one of this work or a potential leak could have accelerated the rate. Lower temperature of UH_3_ preparation, combined with slightly higher temperature of UH_3_ oxidation, were also responsible for the observed higher rate kinetics of Goddard’s results^33^. One of the main findings of Goddard’s work was the strong effect of temperature of UH_3_ formation on the subsequent hydrolysis rate, with measured oxidation rates being one order of magnitude higher for samples prepared at 50 °C, in comparison to those prepared at 160 °C. For the compared rate of that work, they prepared the UH_3_ powder at 80 °C, which is considerably lower formation temperature than used in this present work (240 °C). This had a strong effect on the surface area which was higher (1.24 m^2^.g^−1^), in comparison to this work (~1 m^2^.g^−1^). The three repetitions of hydriding-dehydriding was expected to have resulted in small micron-scale particles with small expected crystallite size^40^. However, a minor possibility is that the relatively high temperature of formation may have resulted in potentially incomplete transformation over the first hydriding sessions. Furthermore, the temperature of dehydriding (500 °C) may have resulted in potential recrystallisation of the metallic particles when these were formed with some associated diffusion bonding between particles^53^. Thus, for our results where the rate was relatively lower, the results are considered reasonable. Additionally, the temperature of the reaction was 5 °C higher (30 °C) than the temperature of reaction used here.

It is not yet clear yet what causes such a drop in kinetics with increasing temperature of UH_3_ formation. Goddard *et al*.^33^ concluded that surface area and crystallite size cannot account for such a difference in the rate. However, if the phase of the hydride switches from α-hydride to β-hydride due to exothermic heating from oxidation, differences in the microstructural features between phases may lead to cracking of the particles, thereby increasing the reactive surface area and accelerating the measured kinetics of diffusion of the oxidising species^33^.

RGA analysis in the UH_3_ + D_2_O experiment provided a closer insight on the early stages of the reaction by tracking the path of D^+^ ions in the gas phase. From this analysis, four observations were highlighted and interpreted:

i. **H**_**2**_ **gas contribution dominated the overall gas pressure in the initial stages of oxidation**. This implied substantial transformation of UH_3_ to UO_2_ through Eq. (3); releasing H_2_ as a by-product.
ii. **The presence of HD in the gas mixture**. Formation of this gas could only occur from combination of H^+^ ions originating from UH_3_ (breakage of the U-H bond) and D^+^ ions originating from dissociation of water. Combination of the H^+^ - D^+^ ions would most likely occur at the hydride-oxide interface, where the H^+^ ions are initially produced. Only through partial dissociation of water (OD^–^D^+^) and diffusion of an OD^-^ entity through the protective oxide, would it be possible for D^+^ ions to reach the hydride-oxide interface and bond with H^+^. Thus, OD^-^ is one (if not the only) oxidising entity in the system. It was not possible to arrive at any further conclusions with regards to the contribution of full dissociation (if any) with the available RGA data. Such a process cannot be excluded from the system. It was also not possible to determine whether HD was forming at the hydride-oxide interface or at the oxide-gas interface. In both instances UO_2_ is a recognised catalyst and has been implicated previously as a reactive surface for gas dissociation and recombination^40^.
iii. **The formation of D**_**2**_ **in the gas reaction products verified the coupling of D**^**+**^ **ions**. This coupling could potentially occur at the gas-oxide or the hydride-oxide interface where D^+^ from dissociation of water or breakage of OD^-^ bond is observed, respectively. Comments from (ii) regarding catalytic behaviour of the oxide also apply.
iv. **The continuous decrease of H**_**2**_ **ratio in the gas products, with increasing oxidation time**. This drop occurs simultaneous to the increase in the HD and D_2_ ratios. Thus, the H_2_ ratio profile line was interrelated with the HD and D_2_ ratio profile lines. This was clearly observed over the entire course of measurement (Fig. 6a–c). In practice, this means that H^+^ ions generated at the hydride-oxide interface will not only recombine with each other but will couple with generated D^+^ ions from the breakage of the OD^-^ bond, at the hydride-oxide interface. The more H^+^ coupled with D^+^ ions, the less residual H^+^ was present to couple with itself to form H_2_. For the D_2_ gas, this means that the gas is produced from both the bonding of D^+^- D^+^ ions at the hydride-oxide interface, but also from recombination of the same ions after partial (or full) dissociation of D_2_O at the gas-oxide interface.

From the above, a mechanism for the oxidative corrosion of UH_3_ with water vapour can be suggested. After newly-formed UH_3_ is produced, very limited oxidation of the surface of the hydride particles is expected to occur over time, especially under UHV conditions. The heat of formation at this stage ranged from 385.4–387 kJ.mol^−1^ ^28,29^, which is significantly lower than that of the UH_3_ + O_2_ system. Once the oxide layer nucleates and covers the hydride particle, the reaction will follow a ‘shrinking core’ or ‘contracting envelope’ model^22,30,32–34^. This was indicated from the decelerated oxidation kinetics over time and the kinetic profile of the reaction (see Eq. (5) and section 1.2.3) Thus, the kinetics will depend on:

- The surface area/particle size of the hydride^33^.
- The growth of the oxide which is controlled by the diffusion of the oxidising entities through the already formed oxide.

The same principles should apply on the gas-oxide interface between H_2_O and UO_2_, as described in the uranium-water oxidation. The presence of HD in the gas products (highlight (ii) above) implies that partial dissociation of D_2_O occurs at the gas-oxide interface, according to the following equation:

6 $${{\boldsymbol{D}}}_{2}{\boldsymbol{O}}\to {{\boldsymbol{D}}}^{+}+{\boldsymbol{O}}{{\boldsymbol{D}}}^{-}$$

Full dissociation (2D^+^ - O^2−^) cannot be excluded from the process, even though partial dissociation should be the dominant process, according to the derived percentage ratios of HD and D_2_ on the generated gases (HD_ratio_ > D_2ratio_). Allen *et al*.^36^ also confirmed the suggestion of a singly charged ion (OD^-^) as the dominating diffusing entity by measuring the difference in the activation energy between uranium and water vapour (38 kJ.mol^−1^) and uranium with oxygen (76.57 kJ.mol^−1^). At the hydride-oxide interface three processes will occur:

7 $${\boldsymbol{U}}{{\boldsymbol{H}}}_{3}\to {\boldsymbol{U}}+3{{\boldsymbol{e}}}^{-}+3{{\boldsymbol{H}}}^{+}$$

8 $${\boldsymbol{U}}\to {{\boldsymbol{U}}}^{4+}+4{{\boldsymbol{e}}}^{-}$$

9 $${\boldsymbol{O}}{{\boldsymbol{D}}}^{-}\to {{\boldsymbol{O}}}^{2-}+{{\boldsymbol{D}}}^{+}$$

Formation of UO_2_ occurs through the following reaction:

10 $${{\boldsymbol{U}}}^{4+}+2\,{{\boldsymbol{O}}}^{2-}\to {\boldsymbol{U}}{{\boldsymbol{O}}}_{2}$$

Three combinations are then possible for the generated H^+^ and D^+^ ions at the hydride-oxide interface. H^+^ ions from Eq. (7) could couple with each other to form H_2_ gas and diffuse out to the headspace as follows:

11 $${{\boldsymbol{H}}}^{+}+{{\boldsymbol{H}}}^{+}+2{{\boldsymbol{e}}}^{-}\to {{\boldsymbol{H}}}_{2({\boldsymbol{g}})}$$

or couple with the D^+^ produced from Eq. (9) to form HD gas as follows:

12 $${{\boldsymbol{H}}}^{+}+{{\boldsymbol{D}}}^{+}+2{{\boldsymbol{e}}}^{-}\to {\boldsymbol{H}}{{\boldsymbol{D}}}_{({\boldsymbol{g}})}$$

It is believed that these two processes are the dominating ones at the hydride-oxide interface. D_2_ formation occurs at the hydride-oxide interface but also at the gas-oxide interface through the same process:

13 $${{\boldsymbol{D}}}^{+}+{{\boldsymbol{D}}}^{+}+2{{\boldsymbol{e}}}^{-}\to {{\boldsymbol{D}}}_{2({\boldsymbol{g}})}$$

with electrons produced at the hydride-oxide interface, partially migrating through the oxide (electron tunnelling) to the gas-oxide interface or incident radiation activating the UO_2_ (as a semi-conductor with 2.2 eV band gap) to create reactive electron hole pairs.

One significant finding of this work was that oxidation of UH_3_ at this temperature and pressure remains incomplete, with the extent of oxidation being ~10% after ~50 days of reaction (UH_3_ + D_2_O experiment). Baker *et al*.^11^ reported a 15–20% of reaction after 14 days at the same conditions, before the rate switches to almost zero kinetics. Goddard *et al*.^33^, reported 27% of UH_3_ conversion after ~20 days of reaction at 22 °C and before the rate slows down considerably. From the same work, with liquid water a ~23% reaction extent was also reported at 30 °C of reaction. Despite the discrepancy in the amount of remaining hydride between studies, this observation of substantial residual hydride after prolonged oxidation in water, ascribed to the forming surface oxide performing as a ‘protective blanket’ and isolating the hydride from further reaction. Under retrieval conditions, breakage of this protective oxide when handling the waste material may trigger a thermal excursion if a substantial bulk mass of UH_3_ becomes exposed to air. In a worst-case scenario (which is highly unlikely) this exothermic phenomenon could reach sufficient temperature to release entrapped volatile fission products, expel fine uranium particles and ignite other flammable materials in the wasteform e.g. magnesium metal. Thus, understanding the conditions under which the material was kept over time, predicting the mass of bulk-UH_3_ formed and then foreseeing the extent that this hydride was oxidised, is highly critical in classifying the risk that a certain batch of ILW may present during retrieval, repackaging, storage or disposal. These results show that bulk-UH_3_ (if initially formed) may still be present in the corroding material after prolonged periods and further confirms the earlier synchrotron work published by Stitt *et al*. who demonstrated the persistence of bulk hydride in a cemented simulant waste form^54^.

In this work, we have examined the oxidation kinetics of UH_3_ powder with water vapour at 25 °C, under saturated conditions. Two experiments were conducted, one with H_2_O and one with D_2_O. RGA analysis of the evolved gases allowed us to gain greater insight into the mechanism of the reaction. From the analysis, it was found that:

i. The kinetics of the reaction show initially robust and continuously decelerating kinetics. This behaviour is indicative of a ‘shrinking core’ or ‘contracting envelope’ type oxidation behaviour.
ii. The kinetics of the quasi-linear decelerating stage were observed to be quite low compared to other works. This was attributed to the formation temperature of the UH_3_ and U particles, respectively (high temperature of UH_3_ formation 240 °C - high dehydriding temperature 500 °C) leading to lower surface area for the UH_3_ powder and potential recrystallisation of the U particles^53^.
iii. The extent of reaction/conversion of UH_3_ was observed to be low: 6.6% after ~32 days for the UH_3_ + H_2_O experiment and ~10% after ~50 days for the UH_3_ + D_2_O experiment. This provides new evidence to bolster the case for the possible long duration persistence of UH_3_ in legacy wastes.
iv. A mechanism for the reaction was suggested by interpreting the gas analysis data from the UH_3_ + D_2_O experiment. The data implicates OH^-^ as the specie with primary responsibility for oxidation, with an associated implication that water vapour is adsorbed and dissociates on the outmost surface of the UO_2_.

## Methods

### Sample preparation

Four uranium metal samples cut from the same parent Magnox-U coupon were used as the precursor material for this work. Extensive characterisation of the samples can be found in^24–27^. The bulk samples were mechanically abraded on all faces using SiC paper (P600) to remove surface grown oxide. One sample was sent to an X-ray diffractometer for surface hydriding *in-situ* using a hot-stage. The remaining three metallic samples were turned to a UH_3_ powder through a standard three-step hydriding-dehydriding process which will be described in the following section. Each sample was reacted separately in a sealed stainless-steel cell, and the UH_3_ powder was prepared under the same pressure-temperature conditions (P = 500 mbar, T = 240 °C) in each case to ensure the consistency of the product materials. Powder preparation was not conducted for each sample at the same time, but only prior to any preliminary analysis or oxidation reaction. One out of the three powder samples was sent for BET surface area analysis, while the other two were used for corrosion reactions. Powdered samples prepared for the oxidation reactions remained in the same reaction cell where the hydriding-dehydriding preparation took place under high dynamic vacuum (1 × 10^−6^ mbar), prior to water vapour introduction. This ensured that no (or only negligible) oxidation would occur on the surface of the newly formed UH_3_ powder. A more detailed description of the experimental procedure will be provided in Section 1.3.4.

### UH_3_ powder preparation (Hydriding - Dehydriding session)

Each bulk uranium sample was placed in a sealed steel cell and connected to a gas control rig. The cell was evacuated down to 1 × 10^−7^ mbar and the temperature increased to 125 °C, while under dynamic vacuum. The sample was left to degas for ~16 h to drive off any adsorbed water. After degassing, the temperature was raised and left to stabilise at 240 °C. This temperature was regarded as ideal for fast phase transformation of the bulk metal sample to hydride powder. It should be noted that only β-UH_3_ is produced at this temperature^52,55,56^. The sample volume was then introduced to 500 mbar of H_2_ gas. The gas was provided by BOC and was stored in a LaNi_5_ bed to further improve purity. Hydriding initiation was observed to occur after a defined induction period (point ‘a’ in Fig. 1). Figure 1 shows the first hydriding session for the sample used for surface area analysis. In the plot, the pressure drop owing to gas absorption by the metal can be observed on ‘a’. After the passing of the accelerating, quasi-linear and decelerating stages of hydride formation (point ‘b’ on Fig. 1), the gas profile line plateaued (point ‘c’ on Fig. 1), signifying that the reaction had completed.

The remaining headspace gas was then fully evacuated and the cell volume isolated and the temperature raised to 500 °C. At this temperature, the hydride powder converts rapidly back to uranium (as a powder) with an associated pressure increase, ascribed to H_2_ gas generation. After no further pressure increase was detected, indicating complete dehydriding of the sample, the volume was evacuated again. The first hydriding-dehydriding session was complete and the temperature was set to 240 °C for re-hydriding of the uranium powder using fresh H_2_ gas. The hydriding-dehydriding cycle was repeated two additional times (three-times in total), preceding the final hydride oxidation experiments. This was done for two main reasons:

i. Non-complete UH_3_ transformation was observed by other investigators after a single cycle of hydriding of uranium under similar conditions^57^.
ii. Negligible change in the surface area of uranium between hydriding-dehydriding reactions was only observed after three hydriding-dehydriding cycles^58^.

Hence, with the preparation above, complete uranium transformation to UH_3_ is expected to be achieved, producing a hydride powder (in each instance) with comparable particle size and surface area.

### Reaction water vapour

Two types of water were used for the oxidising experiments, deionised water for the UH_3_ + H_2_O experiment and deuterated water (D_2_O) for the UH_3_ + D_2_O system. D_2_O was provided by ACROS Organics. Both water beds followed a three-stage freeze-vacuum-melt process.

### Experimental method

Two corrosion experiments were conducted in this work, UH_3_ oxidation with normal water vapour (H_2_O) and another with deuterated water vapour (D_2_O), both under ambient saturated conditions. An identical experimental process was followed for both systems, with the only difference being the source of water providing the oxidising species into the reaction volume (from a bed). Once the UH_3_ powder was prepared (end of 4^th^ hydriding session), the furnace was switched off and the cell was left to cool down to ambient temperature conditions under high dynamic vacuum (<1 × 10^−7^ mbar). The reaction/working volume was then isolated from the turbo-pump and the source water bed was opened for water vapour to be admitted to the cell. Pressure and temperature in the cell were logged every second. Gas composition in the cell was also continually logged using an RGA system (MKS E-Vision instrument). Through the use of isotopically labelled water and by analysing the gases from the start of the reaction, information about the mechanism of the reaction could be derived.

### Analysis techniques

#### BET surface area analysis and XRD analysis

The sample prepared for surface area analysis was sealed and transferred to the BET instrument for measurement. Analysis was performed by making a full 10-point adsorption-desorption isotherm. *In-situ* surface hydriding of the bulk metal was performed using hot-stage XRD to verify that uranium would reliably transform to UH_3_ under the same hydriding conditions used in the gas control rig to make the hydride powder (240 °C, 500 mbar H_2_). The hot-stage XRD set-up is shown in Fig. 2.

#### RGA analysis

RGA analysis of the evolved gases was performed for both oxidation experiments. The RGA analyser was setup only to periodically sample the headspace volume of the cell, by the opening and closing of an automated leak valve. Using an automated valve ensured the same leak magnitude was repeated at every sampling point. The valve was set to open with a 20-min time interval over the first 7 h of reaction. The overall sampling time-period, defined as the period needed for the valve to complete a full opening and closing was ~106 sec. The RGA was set to operate in bar chart mode between 1–50 atomic mass units (amu). The overall time needed for one complete scan was 9 sec. Thus, at least 11 full scans were performed each time the valve was opened. Owing to the very robust and complex nature of the reaction at the early initial stage, the time interval under which gas sampling and analysis was performed was relatively high (t = 20 min). It was imperative that gas evolution over the initial accelerating reaction stage was analysed and recorded, since at this stage of the reaction the mechanism could be verified. Such frequent gas analysis was only performed over the first 7 h of reaction. After the reaction was switched to the quasi-linear decelerating stage, gas analysis was arbitrarily chosen to be performed at ~50 h and ~75 h.

‘Blank’ tests with absence of the uranium powder have been performed to identify if there was any interactions between the water and the stainless steel walls leading to H_2_ formation. Negligible formation of H_2_ has been detected over a long period during the blank tests and was subtracted from our results. Through the use of isotopically labelled water and by analysing the gases at the early initial stages, information about the mechanism of the reaction could be derived.

## Data Availability

The datasets generated during and/or analysed during the current study are available from the corresponding author on reasonable request.
